# Supplementary material for: D‐Aspartate treatment attenuates myelin damage and stimulates myelin repair
Source: EMBO Mol Med. 2018 Dec 17;11(1):e9278. doi: 10.15252/emmm.201809278 (PMC6328990; doi:10.15252/emmm.201809278)
Supplement: Supplementary file 1 — Appendix [file EMMM-11-e9278-s001.pdf]

APPENDIX

Complete statistical results and exact P-values for each figure.

Table of contents:

page

Appendix Table S1 2

Appendix Table S2 3

Appendix Table S3 4

Appendix Table S4 5

Appendix Table S5 6

Appendix Table S6 7

Appendix Table S7 8

Appendix Table S8 9

Appendix Table S9 10

Appendix Table S10 11

Appendix Table S1

FIGURE	COMPARISON	P VALUE	POST HOC TEST
**1A, left panel**	ctrl vs D-Asp 10 µM	ns	**Tukey**
	ctrl vs D-Asp 100 µM	0.0431	
	ctrl vs D-Asp 200 µM	< 0.0001	
	ctrl vs PMA	< 0.0001	
**1A, right panel**	ctrl vs D-Asp 10 µM	ns	**Tukey**
	ctrl vs D-Asp 100 µM	0.0024	
	ctrl vs D-Asp 200 µM	0.0005	
	ctrl vs PMA	< 0.0001	
**1B**	ctrl vs D-Asp 100 µM	0.0224	**Bonferroni**
	ctrl vs D-Asp 200 µM	0.0002	
	ctrl vs PMA	0.0043	
**1C**	ctrl vs D-Asp (3d)	0.0438	**Student's *t*-tests**
**1F, f**	ctrl vs D-Asp (Low)	0.001	**Student's *t*-tests**
	ctrl vs D-Asp (Medium)	0.0083	**Student's *t*-tests**
	ctrl vs D-Asp (M-High)	0.0001	**Student's *t*-tests**
**1F, g**	ctrl vs D-Asp (MAG+ cells)	0.0002	**Student's *t*-tests**

Appendix Table S2

FIGURE	COMPARISON	P VALUE	POST HOC TEST
**2C**	ctrl vs D-Asp	< 0.0001	**Student's *t*-tests**
**2D**	ctrl vs D-Asp	0.0457	**Student's *t*-tests**
**2G**	ctrl vs LPC (6dpl)<br>LPC vs LPC + D-Asp (6dpl)	0.0248<br>0.0025	**Bonferroni**
**2H**	ctrl vs LPC (6dpl)<br>LPC vs LPC + D-Asp (6dpl)	0.0033<br>< 0.0001	**Bonferroni**
**2J**	ctrl vs LPC (10dpl)<br>LPC vs LPC + D-Asp (10dpl)	< 0.0001<br>< 0.0001	**Bonferroni**

Appendix Table S3

FIGURE	COMPARISON	P VALUE	POST HOC TEST
**3A. left panel**	ctrl vs D-Asp<br>D-Asp vs MK-801	0.0012<br>0.0096	**Tukey**
**3A, right panel**	ctrl vs D-Asp<br>D-Asp vs D-Asp + BED<br>D-Asp vs D-Asp + YM244769	0.0001<br>0.0009<br>0.0041	**Tukey**
**3B, left panel**	ctrl vs D-Asp<br>D-Asp vs MK-801	0.0002<br>0.0002	**Tukey**
**3B, right panel**	ctrl vs D-Asp<br>D-Asp vs D-Asp + BED<br>D-Asp vs D-Asp + YM244769	0.0006<br>0.0024<br>0.0104	**Tukey**
**3C, m**	D-Asp vs D-Asp + MK-801<br>D-Asp vs D-Asp + BED<br>D-Asp vs D-Asp + YM244769	0.0341<br>< 0.0001<br>< 0.0001	**Bonferroni**
**3C, n**	D-Asp vs D-Asp + MK-801<br>D-Asp vs D-Asp+ BED<br>D-Asp vs D-Asp + YM244769	0.0054<br>< 0.0001<br>< 0.0001	**Bonferroni**
**3D**	ctrl vs D-Asp 10 µM<br>ctrl vs D-Asp 100 µM<br>ctrl vs D-Asp 200 µM<br>ctrl vs PMA	0.0452<br>0.0015<br>0.0003<br>< 0.0001	**Tukey**
**3E**	ctrl vs D-Asp<br>D-Asp vs MK-801	0.0107<br>0.0369	**Tukey**

Appendix Table S4

FIGURE	COMPARISON	P VALUE	POST HOC TEST
**4A**	ctrl vs D-Asp<br>D-Asp vs D-Asp + MK-801<br>D-Asp vs D-Asp + APV<br>D-Asp + MK-801 vs D-Asp + APV	< 0.0001<br>< 0.0001<br>< 0.0001<br>ns	**Bonferroni**
**4a**	ctrl vs D-Asp<br>D-Asp vs D-Asp + MK-801<br>D-Asp vs D-Asp + APV<br>D-Asp + MK-801 vs D-Asp + APV	< 0.0001<br>< 0.0001<br>< 0.0001<br>ns	**Bonferroni**
**4B**	ctrl vs D-Asp<br>D-Asp vs D-Asp + MK-801<br>D-Asp vs D-Asp + APV<br>D-Asp + MK vs D-Asp + APV	< 0.0001<br>< 0.0001<br>< 0.0001<br>ns	**Bonferroni**
**4b**	ctrl vs D-Asp<br>D-Asp vs D-Asp + MK-801<br>D-Asp vs D-Asp + APV<br>D-Asp + MK-801 vs D-Asp + APV	< 0.0001<br>< 0.0001<br>< 0.0001<br>ns	**Bonferroni**
**4C**	ctrl vs D-Asp<br>D-Asp vs D-Asp + BED<br>D-Asp vs D-Asp + YM244769	< 0.0001<br>< 0.0001<br>< 0.0001	**Bonferroni**
**4c**	ctrl vs D-Asp<br>D-Asp vs D-Asp + BED<br>D-Asp vs D-Asp + YM244769<br>D-Asp + BED vs D-Asp + YM244769	< 0.0001<br>< 0.0001<br>< 0.0001<br>ns	**Bonferroni**
**4D**	ctrl vs D-Asp<br>D-Asp vs D-Asp + BED<br>D-Asp vs D-Asp + YM244769<br>D-Asp + BED vs D-Asp + YM244769	< 0.0001<br>< 0.0001<br>< 0.0001<br>ns	**Bonferroni**
**4d**	ctrl vs D-Asp<br>D-Asp vs D-Asp + BED<br>D-Asp vs D-Asp + YM244769<br>D-Asp + BED vs D-Asp + YM244769	< 0.0001<br>< 0.0001<br>< 0.0001<br>ns	**Bonferroni**
**4E**	ctrl vs siCtl<br>ctrl + siCtl vs D-Asp + siCtl<br>D-Asp + siCtl vs D-Asp + siNCX3	ns<br>< 0.0001<br>< 0.0001	**Bonferroni**
**4e**	ctrl vs siCtl<br>ctrl + siCtl vs D-Asp + siCtl<br>D-Asp + siCtl vs D-Asp + siNCX3	ns<br>< 0.0001<br>< 0.0001	**Bonferroni**
**4F**	ctrl vs ncx3+/+<br>ncx3+/+ vs ncx3+/-<br>ncx3+/+ vs ncx3-/-	< 0.0001<br>< 0.0001<br>0.006	**Bonferroni**
**4f**	ctrl vs ncx3+/+<br>ncx3+/+ vs ncx3+/-<br>ncx3+/+ vs ncx3-/-	< 0.0001<br>ns<br>0.006	**Bonferroni**

Appendix Table S5

FIGURE	COMPARISON	P VALUE	POST HOC TEST
**5A**	1μM AMPA vs 10μM AMPA	<0.001	**Bonferroni**
	1μM AMPA vs 100μM AMPA	<0.001	
	10μM AMPA vs 100μM AMPA	<0.001	
**5C**	AMPA (I) vs AMPA + DNQX	<0.001	**Bonferroni**
	AMPA (II) vs AMPA + DNQX	<0.001	
	AMPA (IV) vs AMPA + DNQX	<0.001	
**5D**	D-Asp (I) vs D-Asp + DNQX	<0.001	**Bonferroni**
	D-Asp (II) vs D-Asp + DNQX	<0.001	
	D-Asp (IV) vs D-Asp + DNQX	<0.001	
**5G**	AMPA (I) vs AMPA + DNQX	<0.001	**Bonferroni**
	AMPA (II) vs AMPA (IV)	<0.001	
	AMPA (II) vs AMPA + DNQX	0.002	
	AMPA (IV) vs AMPA + DNQX	<0.001	
	AMPA (IV) vs AMPA (I)	0.044	
**5H**	D-Asp (I) vs D-Asp + DNQX	<0.001	**Bonferroni**
	D-Asp (I) vs D-Asp + DNQX + MK-801	<0.001	
	D-Asp (I) vs D-Asp + DNQX + MK-801 + PDC	<0.001	
	D-Asp (II) vs D-Asp + DNQX	<0.001	
	D-Asp (II) vs D-Asp + DNQX + MK-801	<0.001	
	D-Asp (II) vs D-Asp + DNQX + MK-801 + PDC	<0.001	
	D-Asp + DNQX vs D-Asp (VI)	<0.001	
	D-Asp+DNQX+MK-801 vs D-Asp+DNQX+MK-801+PDC	0.023	
	D-Asp + DNQX + MK-801 + PDC vs D-Asp (VI)	<0.001	
	D-Asp (VI) vs D-Asp (I)	0.001	
	D-Asp (VI) vs D-Asp (II)	<0.001	
	D-Asp + DNQX + MK-801 vs D-Asp (II)	<0.001	

Appendix Table S6

FIGURE	COMPARISON	P VALUE	POST HOC TEST
**6B, left panel**	ctrl vs cpz (1d)<br>cpz vs D-Asp (1d)	0.0018<br>0.0399	**Bonferroni**
**6B, right panel**	ctrl vs cpz<br>cpz vs cpz + D-Asp	0.0002<br>0.0281	**Bonferroni**
**6C, left panel**	ctrl vs cpz (1d)<br>cpz vs D-Asp (1d)	0.0026<br>0.0371	**Bonferroni**
**6C, right panel**	ctrl vs cpz<br>cpz vs cpz + D-Asp	< 0.0001<br>0.0018	**Bonferroni**
**6D, left panel**	ctrl vs vehicle (3d)<br>cpz vs D-Asp (II) (3d)	0.0038<br>0.0303	**Bonferroni**
**6D, middle panel**	ctrl vs vehicle<br>ctrl vs cpz+D-Asp (I)	< 0.0001<br>< 0.0001	**Bonferroni**
**6D, right panel**	ctrl vs vehicle<br>cpz vs cpz+D-Asp (II)	< 0.0001<br>< 0.0001	**Bonferroni**
**6E, left panel**	cpz vs cpz + D-Asp (2d)	0.0035	**Bonferroni**
	ctrl vs cpz (3d)<br>cpz vs cpz + D-Asp (3d)	< 0.0001<br>0.0006	**Bonferroni**
**6E, right panel**	ctrl vs cpz<br>ctrl vs cpz + D-Asp	0.0005<br>0.0172	**Bonferroni**
**6F, left panel**	ctrl vs cpz (3d)<br>cpz vs cpz + D-Asp (3d)	0.0178<br>0.039	**Bonferroni**
**6F, right panel**	ctrl vs cpz<br>ctrl vs cpz + D-Asp	< 0.0001<br>0.0132	**Bonferroni**
**6G, left panel**	ctrl vs vehicle (1d)<br>ctrl vs D-Asp (II) (1d)<br>vehicle vs D-Asp (II) (1d)	< 0.0001<br>0.0153<br>0.0045	**Bonferroni**
	ctrl vs vehicle (2d)<br>vehicle vs D-Asp (II) (2d)	0.0356<br>0.0079	**Bonferroni**
	ctrl vs vehicle (3d)<br>vehicle vs D-Asp (II) (3d)	0.0061<br>0.0394	**Bonferroni**
**6G, right panel**	ctrl vs vehicle<br>vehicle vs D-Asp (II)	< 0.0001<br>< 0.0001	**Bonferroni**

Appendix Table S7

FIGURE	COMPARISON	P VALUE	POST HOC TEST
**7A, j**	ctrl vs cpz<br>cpz vs cpz + D-Asp	< 0.0001<br>< 0.0001	**Bonferroni**
**7A, k**	ctrl vs cpz<br>cpz vs cpz + D-Asp	<0.05<br><0.05	**Newman-Keuls**
**7B**	ctrl vs cpz<br>ctrl vs D-Asp<br>cpz vs D-Asp	< 0.0001<br>0.0303<br>< 0.0001	**Bonferroni**
**7C, j**	ctrl vs cpz<br>cpz vs cpz + D-Asp	< 0.0001<br>0.0002	**Bonferroni**
**7C, k**	ctrl vs cpz<br>cpz vs cpz + D-Asp	< 0.0001<br>0.0062	**Bonferroni**
**7D, middle panel**	ctrl vs cpz<br>ctrl vs cpz + D-Asp	0.028<br>0.0365	**Bonferroni**
**7D, right panel**	ctrl vs cpz<br>cpz vs cpz + D-Asp	0.0045<br>0.0107	**Bonferroni**

Appendix Table S8

FIGURE	COMPARISON	P VALUE	POST HOC TEST
**8A, k, left panel**	ctrl vs cpz (Olig2+ cells)<br>ctrl vs cpz + D-Asp (Olig2+ cells)<br>cpz vs cpz + D-Asp (Olig2+ cells)	≤ 0.0001<br>≤ 0.0001<br>≤ 0.05	**Newman-Keuls**
	ctrl vs cpz (Olig2+/CC1+ cells)<br>ctrl vs cpz + D-Asp (Olig2+/CC1+ cells)<br>cpz vs cpz + D-Asp (Olig2+/CC1+ cells)	≤ 0.0001<br>≤ 0.0001<br>≤ 0.05	**Newman-Keuls**
**8A, k, right panel**	ctrl vs vehicle (Olig2+cells)<br>ctrl vs cpz + D-Asp (Olig2+ cells)<br>vehicle vs cpz + D-Asp (Olig2+ cells)	≤ 0.0001<br>≤ 0.001<br>≤ 0.01	**Newman-Keuls**
	ctrl vs vehicle (Olig2+/CC1+ cells)<br>ctrl vs cpz + D-Asp (Olig2+/CC1+ cells)<br>vehicle vs cpz + D-Asp (Olig2+/CC1+ cells)	≤ 0.0001<br>≤ 0.0001<br>≤ 0.01	**Newman-Keuls**
**8C**	cpz vs cpz + D-Asp	0.0004	**Student's *t*-tests**
**8E**	vehicle vs D-Asp (0.2 μm axons)<br>vehicle vs D-Asp (0.3 μm axons)<br>vehicle vs D-Asp (0.4 μm axons)<br>vehicle vs D-Asp (0.5 μm axons)<br>vehicle vs D-Asp (0.6 μm axons)<br>vehicle vs D-Asp (0.7 μm axons)	0.0097<br>0.0005<br>< 0.0001<br>< 0.0001<br>0.0157<br>< 0.0001	**Student's *t*-tests**<br>**Student's *t*-tests**<br>**Student's *t*-tests**<br>**Student's *t*-tests**<br>**Student's *t*-tests**<br>**Student's *t*-tests**
**8F**	vehicle vs D-Asp (0.2 μm axons)<br>vehicle vs D-Asp (0.3 μm axons)<br>vehicle vs D-Asp (0.4 μm axons)	0.0347<br>0.0289<br>0.0457	**Student's *t*-tests**<br>**Student's *t*-tests**<br>**Student's *t*-tests**

Appendix Table S9

FIGURE	COMPARISON	P VALUE	POST HOC TEST
**EV1A**	ctrl vs AMPA<br>AMPA vs AMPA + DNQX	< 0.0001<br>< 0.0001	**Bonferroni**
**EV1B**	ctrl vs D-Asp<br>D-Asp vs D-Asp + DNQX	< 0.0001<br>< 0.0001	**Bonferroni**
**EV1B, b**	ctrl vs D-Asp<br>D-Asp vs D-Asp + DNQX	< 0.0001<br>< 0.0001	**Bonferroni**
**EV1C**	ctrl vs D-Asp<br>D-Asp vs D-Asp + CNQX	< 0.0001<br>< 0.0001	**Bonferroni**
**EV1C, c**	ctrl vs D-Asp<br>D-Asp vs D-Asp + CNQX	< 0.0001<br>< 0.0001	**Bonferroni**
**EV1D**	ctrl vs D-Asp<br>D-Asp vs D-Asp + PDC	< 0.0001<br>< 0.0001	**Bonferroni**
**EV1D, d**	ctrl vs D-Asp<br>D-Asp vs D-Asp + PDC	< 0.0001<br>< 0.0001	**Bonferroni**

Appendix Table S10

FIGURE	COMPARISON	P VALUE	POST HOC TEST
**EV2A, left panel**	ctrl vs cpz (3d)<br>cpz vs cpz + D-Asp (3d)	0.0226<br>0.031	**Bonferroni**
**EV2A, right panel**	ctrl vs cpz	0.0181	**Bonferroni**
**EV2B, right panel**	ctrl vs cpz<br>cpz vs D-Asp	0.0058<br>0.0414	**Bonferroni**
**EV2C, left panel**	ctrl vs cpz (2d)<br>cpz vs D-Asp (II) (2d)	0.03<br>0.0409	**Bonferroni**
	ctrl vs cpz (3d)<br>cpz vs D-Asp (II) (3d)	0.0114<br>0.01	**Bonferroni**
**EV2C, right panel**	ctrl vs cpz<br>cpz vs D-Asp (II)	0.0005<br>0.0182	**Bonferroni**
