# Supplementary material for: D‐Aspartate treatment attenuates myelin damage and stimulates myelin repair
Source: EMBO Mol Med. 2018 Dec 17;11(1):e9278. doi: 10.15252/emmm.201809278 (PMC6328990; doi:10.15252/emmm.201809278)
Supplement: Supplementary file 2 — Expanded View Figures PDF [file EMMM-11-e9278-s002.pdf]

Expanded View Figures

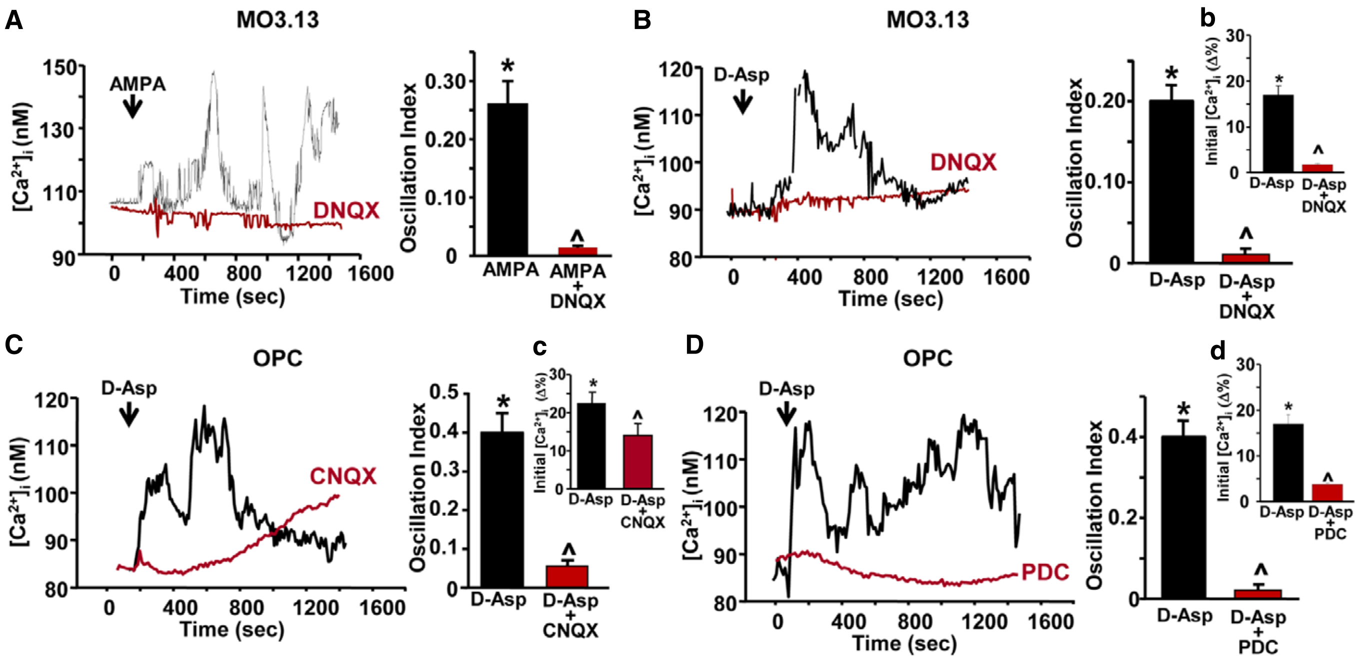

Figure EV1. Effect of AMPA receptors and glutamate transporters blockade on D-Asp elicited $[Ca^{2+}]_i$ increase in oligodendrocyte precursors.

A Left: Superimposed single-cell traces representative of the effect of 10 μM AMPA on $[Ca^{2+}]_i$ detected in MO3.13 cells in the absence or in the presence of 10 μM DNQX. Right: Quantification of the oscillation index in MO3.13 cells after 10 μM AMPA exposure in the absence or in the presence of 10 μM DNQX.

B Left: Superimposed single-cell traces representative of the effect of 100 μM D-Asp on $[Ca^{2+}]_i$ detected in MO3.13 cells in the absence or in the presence of 10 μM DNQX. Right: Quantification of the oscillation index in MO3.13 progenitors after 100 μM D-Asp exposure in the absence or in the presence of 10 μM DNQX. (b) Quantification of the initial $[Ca^{2+}]_i$ increase elicited by D-Asp in M03.13 cells measured as Δ% of peak versus basal values in the absence or in the presence of 10 μM DNQX.

C Left: Superimposed single-cell traces representative of the effect of 100 μM D-Asp on $[Ca^{2+}]_i$ detected in OPC in the absence or in the presence of 25 μM CNQX. CNQX was preincubated 5 min before registration. Right: Quantification of the oscillation index in OPC after 100 μM D-Asp exposure in the absence or in the presence of 25 μM CNQX. (c) Quantification of the initial $[Ca^{2+}]_i$ increase elicited by D-Asp in OPC measured as Δ% of peak versus basal values in the absence or in the presence of 25 μM CNQX.

D Left: Superimposed single-cell traces representative of the effect of 100 μM D-Asp on $[Ca^{2+}]_i$ detected in OPC in the absence or in the presence of 20 μM L-*trans*-pyrrolidine-2,4-dicarboxylic acid (PDC). PDC was preincubated 5 min before registration. Right: Quantification of the oscillation index in OPC after 100 μM D-Asp exposure in the absence or in the presence of 20 μM PDC. (d) Quantification of the initial $[Ca^{2+}]_i$ increase elicited by D-Asp in OPC measured as Δ% of peak versus basal values in the absence or in the presence of 20 μM PDC.

Data information: The values represent the mean ± SEM from three independent experimental sessions. Level of significance was determined by using: in (A) one-way ANOVA $P < 0.001$ followed by Bonferroni *post hoc* test, *$P < 0.05$ versus control (basal value), ^$P < 0.05$ versus AMPA. Data are reported as mean of 15 cells in each group, $n = 3$ biological replicates; (B and b) one-way ANOVA $P < 0.001$ followed by Bonferroni *post hoc* test, *$P < 0.05$ versus control (basal value), ^$P < 0.05$ versus D-Asp. Data are reported as mean of 22–30 cells in each group, $n = 3$ biological replicates; (C and c) one-way ANOVA $P < 0.001$ and $P = 0.023$, respectively, followed by Bonferroni *post hoc* test, *$P < 0.05$ versus control (basal value); ^$P < 0.05$ versus D-Asp. Data are reported as mean of 18–20 cells in each group, $n = 3$ biological replicates; (D and d) one-way ANOVA $P < 0.001$ followed by Bonferroni *post hoc* test, *$P < 0.05$ versus control (basal value); ^$P < 0.05$ versus D-Asp. Data are reported as mean of 20 cells in each group, $n = 3$ biological replicates. See the exact *P*-values from comparisons tests in Appendix Table S9.

© 2018 The Authors

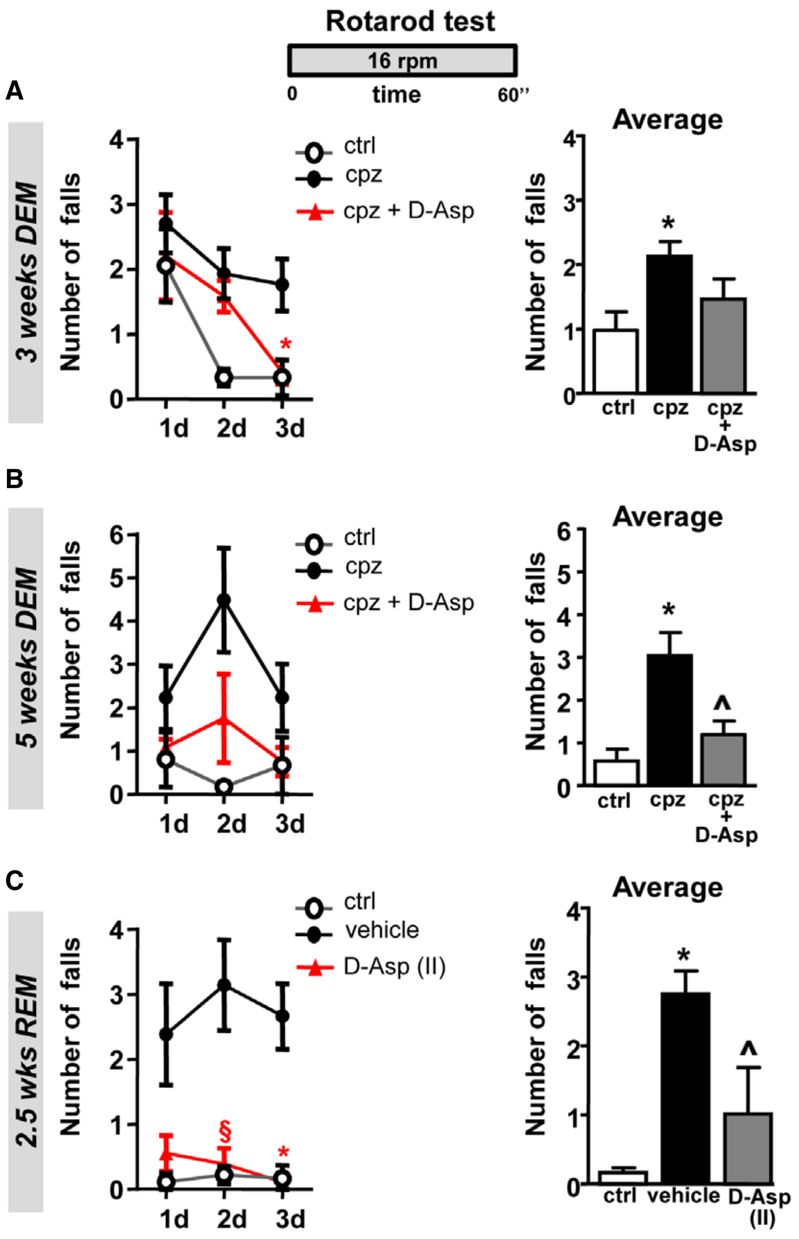

Figure EV2. Effects of D-Asp treatment on motor coordination performance in fixed rotarod test.

A, B Number of falls during daily training in fixed-speed rotarod test (left panel, averaged across three trials per day) and average of falls over 3 consecutive days (right panel) recorded in control (open circles), cuprizone- (filled black circles), and D-Asp-treated (filled red triangles) mice at 3 weeks (A) and 5 weeks (B) of cuprizone feeding.

C Number of falls during daily training in fixed-speed rotarod test (left panel, averaged across three trials per day) and average of falls over 3 consecutive days (right panel) recorded after 2.5 weeks of remyelination in control (open circles), cuprizone- (filled black circles), and D-Asp-treated mice. D-Asp (II) (filled red triangles) refers to the group of mice which received D-Asp during the last week of cuprizone feeding and for 2.5 additional weeks after cuprizone withdrawal. DEM, demyelination; REM, remyelination.

Data information: The values represent the means ± SEM (n = 6 mice for each group). Level of significance was determined by using: in (A–C) left panels (daily training), for each day (d) one-way ANOVA P = 0.0067 (A), P = 0.0111 (C, 2 day), and P = 0.0029 (C, 3 day) followed by Bonferroni *post hoc* test, $^{\S}P < 0.05$ versus cpz at day 2; $^{*}P < 0.05$ versus cpz at day 3 (n = 6 mice for each group). In (A–C) right panels (average): one-way ANOVA P = 0.0165 (A), P = 0.0025 (B), P = 0.0003 (C), followed by Bonferroni *post hoc* test, $^{*}P < 0.05$ versus control; $^{\wedge}P < 0.05$ versus cpz or vehicle (veh). See the exact P-values from comparisons tests in Appendix Table S10.
