# Supplementary figures and images for: D‐Aspartate treatment attenuates myelin damage and stimulates myelin repair
Source: EMBO Mol Med. 2018 Dec 17;11(1):e9278. doi: 10.15252/emmm.201809278 (PMC6328990; doi:10.15252/emmm.201809278)

# Figure 1B

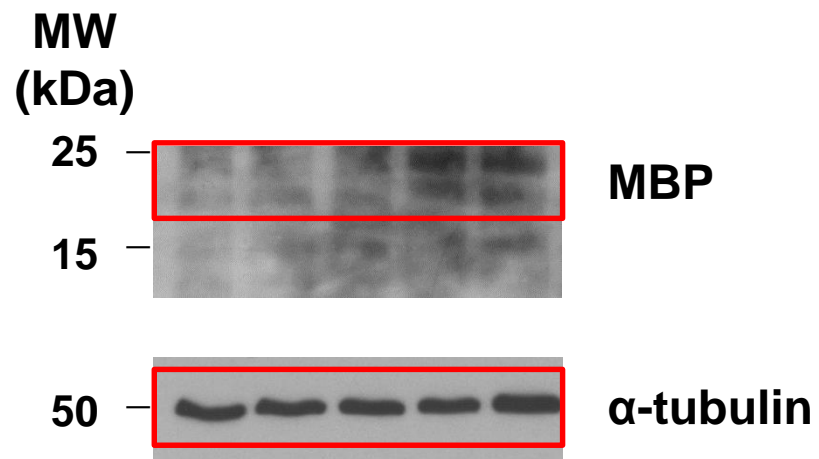

Supplement: Supplementary file 4 — Source Data for Figure 1B [file EMMM-11-e9278-s003.pdf]

# Figure 2D

Not denaturated samples

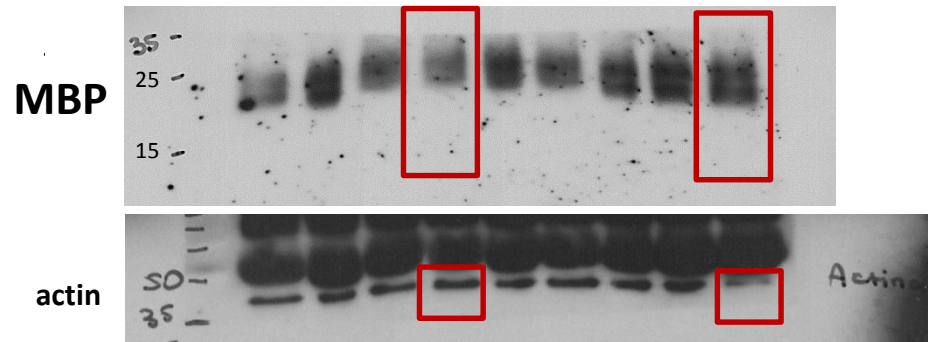

Denaturated samples

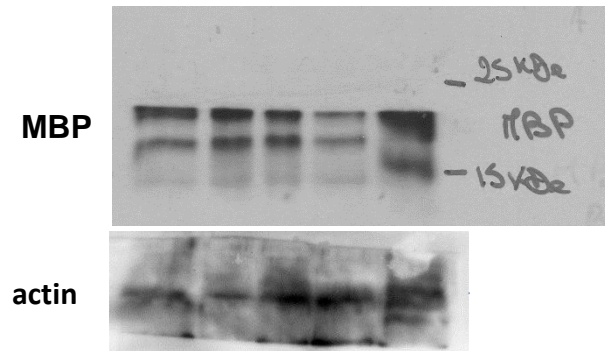

# Figure 2G

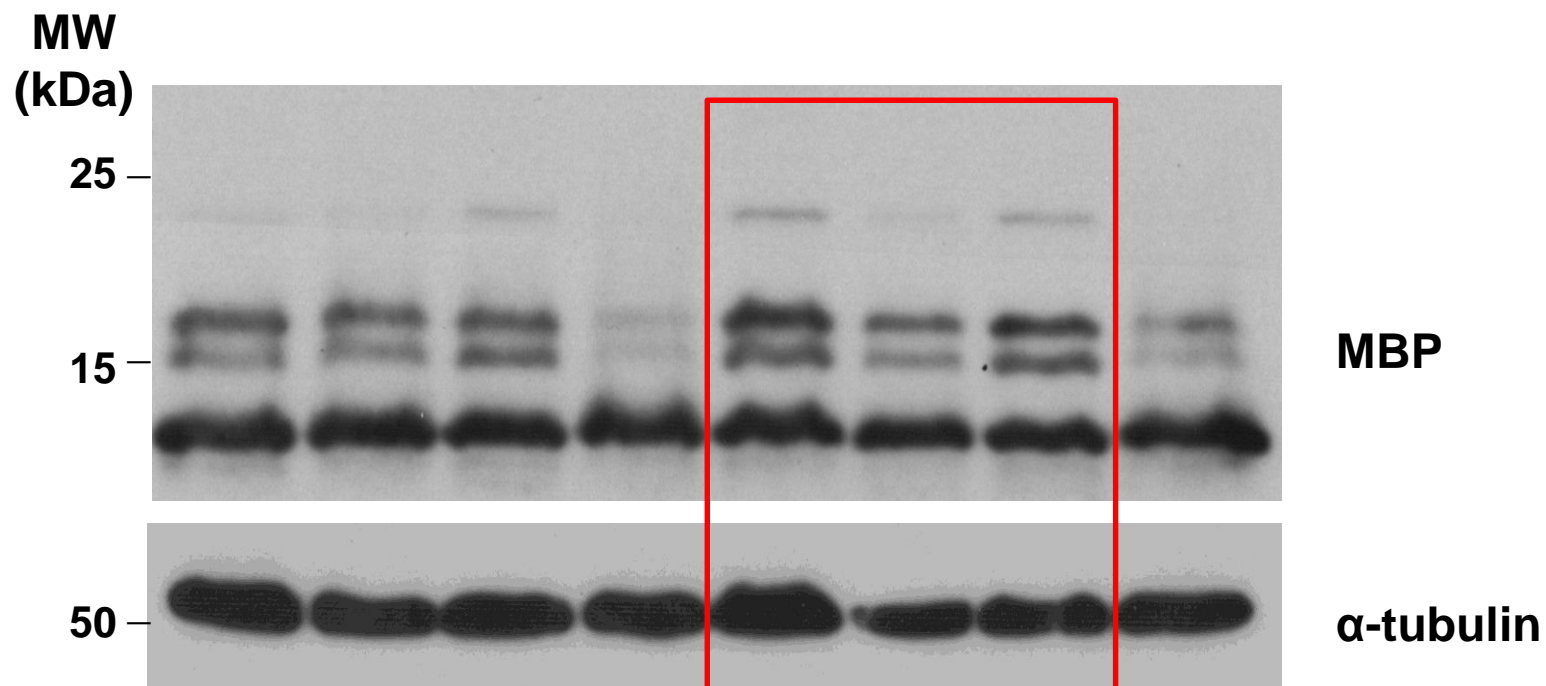

Supplement: Supplementary file 5 — Source Data for Figure 2D and G [file EMMM-11-e9278-s004.pdf]

Figure 7B

MW  
(kDa)

25

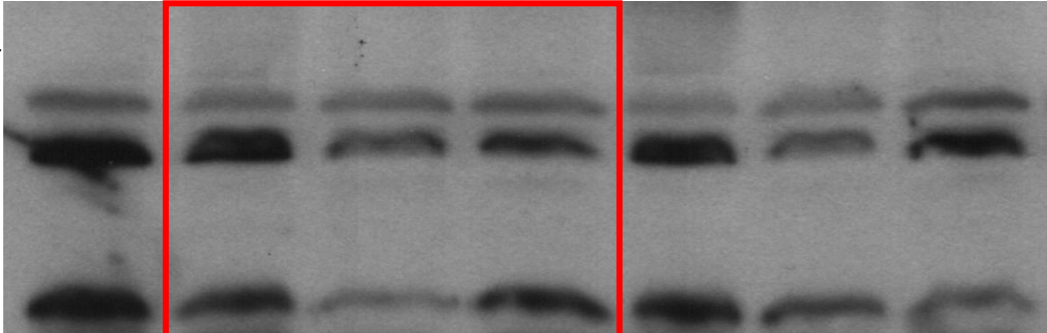

MBP

50

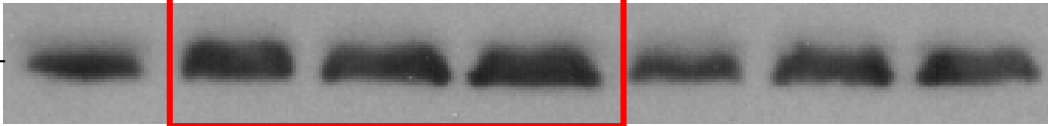

$\alpha$ -tubulin

Figure 7C

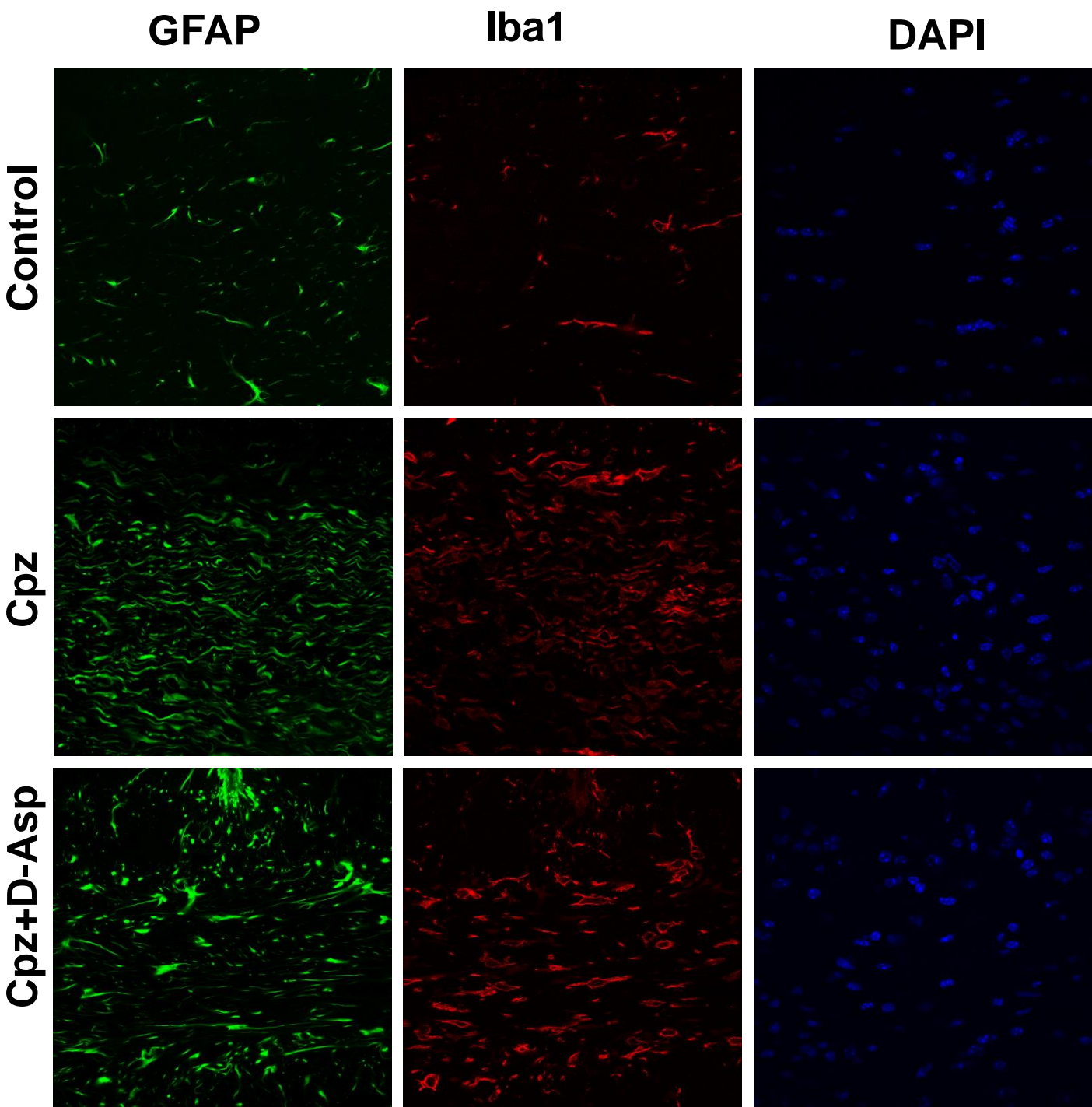

Figure 7D

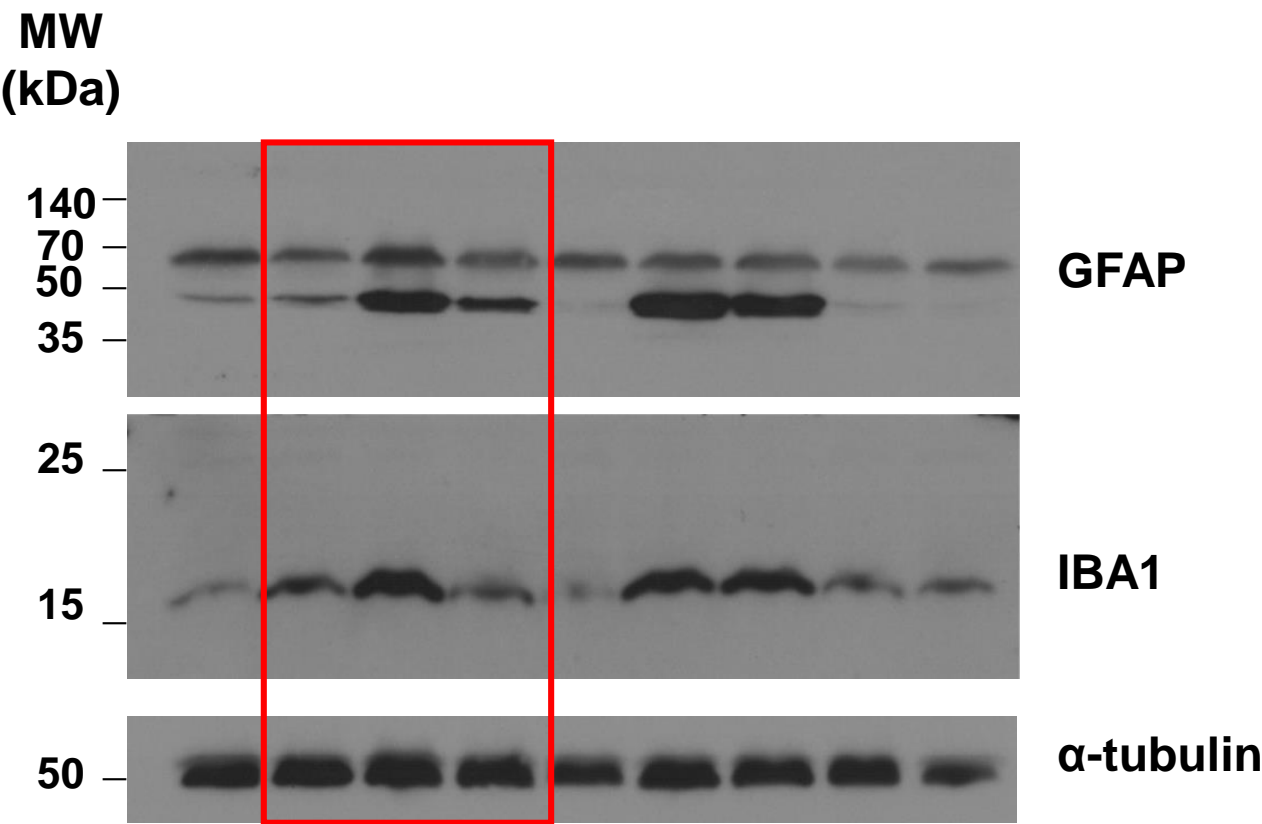

Supplement: Supplementary file 6 — Source Data for Figure 7B–D [file EMMM-11-e9278-s005.pdf]

**Figure 8A**

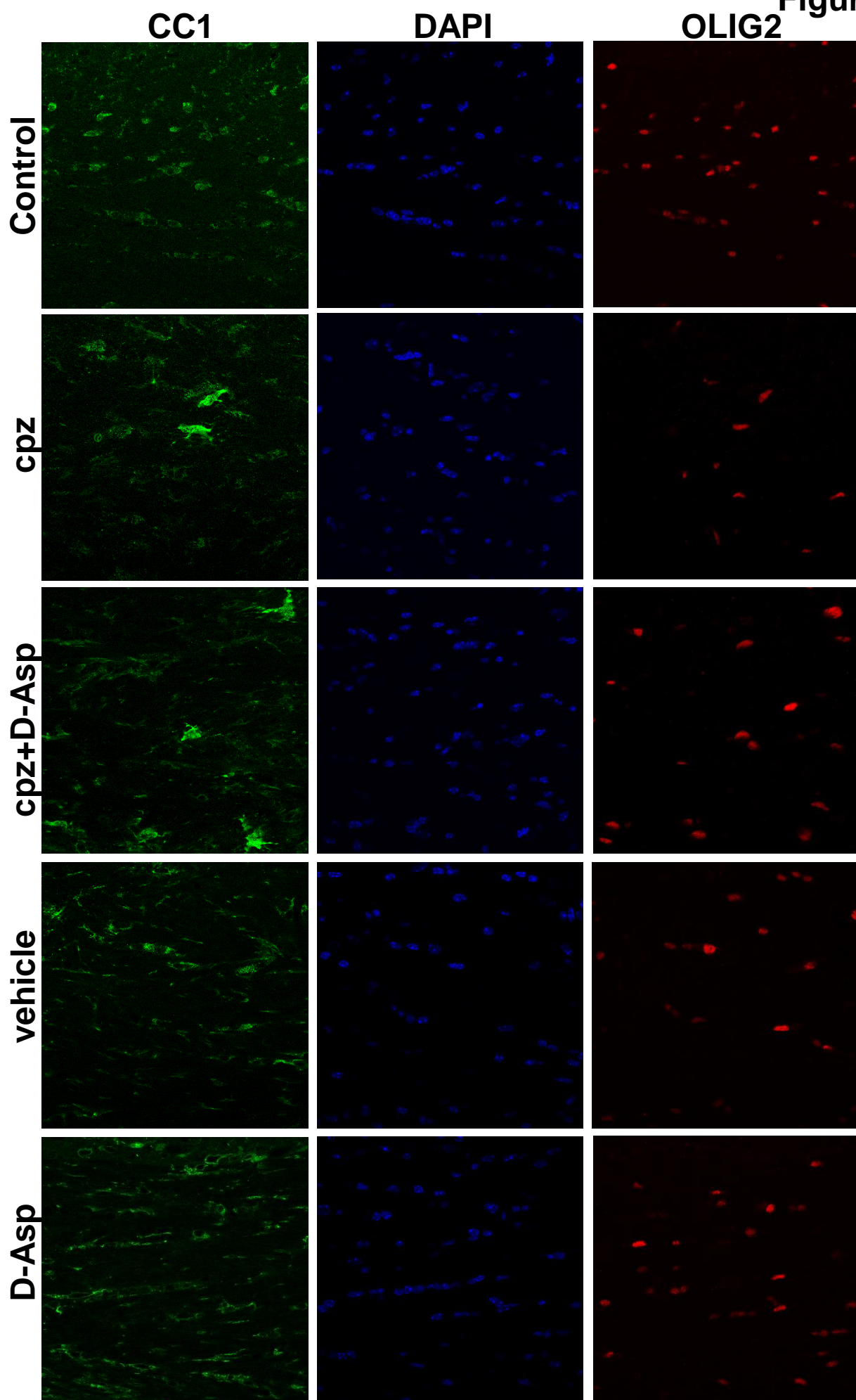

Supplement: Supplementary file 7 — Source Data for Figure 8A [file EMMM-11-e9278-s006.pdf]
